# Patient Use and Clinical Practice Patterns of Remote Cardiology Clinic Visits in the Era of COVID-19

**DOI:** 10.1001/jamanetworkopen.2021.4157

**Published:** 2021-04-05

**Authors:** Neal Yuan, Joshua M. Pevnick, Patrick G. Botting, Yaron Elad, Shaun J. Miller, Susan Cheng, Joseph E. Ebinger

**Affiliations:** 1 Smidt Heart Institute, Cedars-Sinai Medical Center, Los Angeles, California; 2 Department of Medicine, Cedars-Sinai Medical Center, Los Angeles, California; 3 Division of Informatics, Department of Biomedical Sciences, Cedars-Sinai Medical Center, Los Angeles, California

## Abstract

**Importance:**

The COVID-19 pandemic has led to an unprecedented shift in ambulatory cardiovascular care from in-person to remote visits.

**Objective:**

To understand whether the transition to remote visits is associated with disparities in patient use of care, diagnostic test ordering, and medication prescribing.

**Design, Setting, and Participants:**

This cross-sectional study used electronic health records data for all ambulatory cardiology visits at an urban, multisite health system in Los Angeles County, California, during 2 periods: April 1, 2019, to December 31, 2019 (pre-COVID) and April 1 to December 31, 2020 (COVID-era). Statistical analysis was performed from January to February 2021.

**Exposure:**

In-person or remote ambulatory cardiology clinic visit at one of 31 during the pre-COVID period or COVID-era period.

**Main Outcomes and Measures:**

Comparison of patient characteristics and frequencies of medication ordering and cardiology-specific testing across 4 visit types (pre-COVID in-person (reference), COVID-era in-person, COVID-era video, COVID-era telephone).

**Results:**

This study analyzed data from 87 182 pre-COVID in-person, 74 498 COVID-era in-person, 4720 COVID-era video, and 10 381 COVID-era telephone visits. Across visits, 79 572 patients were female (45.0%), 127 080 patients were non-Hispanic White (71.9%), and the mean (SD) age was 68.1 (17.0) years. Patients accessing COVID-era remote visits were more likely to be Asian, Black, or Hispanic individuals (24 934 pre-COVID in-person visits [28.6%] vs 19 742 COVID-era in-person visits [26.5%] vs 3633 COVID-era video visits [30.4%] vs 1435 COVID-era telephone visits [35.0%]; *P* < .001 for all comparisons), have private insurance (34 063 pre-COVID in-person visits [39.1%] vs 25 474 COVID-era in-person visits [34.2%] vs 2562 COVID-era video visits [54.3%] vs 4264 COVID-era telephone visits [41.1%]; *P* < .001 for COVID-era in-person vs video and COVID-era in-person vs telephone), and have cardiovascular comorbidities (eg, hypertension: 37 166 pre-COVID in-person visits [42.6%] vs 31 359 COVID-era in-person visits [42.1%] vs 2006 COVID-era video visits [42.5%] vs 5181 COVID-era telephone visits [49.9%]; *P* < .001 for COVID-era in-person vs telephone; and heart failure: 14 319 pre-COVID in-person visits [16.4%] vs 10 488 COVID-era in-person visits [14.1%] vs 1172 COVID-era video visits [24.8%] vs 2674 COVID-era telephone visits [25.8%]; *P* < .001 for COVID-era in-person vs video and COVID-era in-person vs telephone). After adjusting for patient and visit characteristics and in comparison with pre-COVID in-person visits, during video and telephone visits, clinicians had lower odds of ordering any medication (COVID-era in-person: odds ratio [OR], 0.62 [95% CI, 0.60-0.64], COVID-era video: OR, 0.22 [95% CI, 0.20-0.24]; COVID-era telephone: OR, 0.14 [95% CI, 0.13-0.15]) or tests, such as electrocardiograms (COVID-era in-person: OR, 0.60 [95% CI, 0.58-0.62]; COVID-era video: OR, 0.03 [95% CI, 0.02-0.04]; COVID-era telephone: OR, 0.02 [95% CI, 0.01-0.03]) or echocardiograms (COVID-era in-person: OR, 1.21 [95% CI, 1.18-1.24]; COVID-era video: OR, 0.47 [95% CI, 0.42-0.52]; COVID-era telephone: OR, 0.28 [95% CI, 0.25-0.31]).

**Conclusions and Relevance:**

Patients who were Asian, Black, or Hispanic, had private insurance, and had at least one of several cardiovascular comorbidities used remote cardiovascular care more frequently in the COVID-era period. Clinician ordering of diagnostic testing and medications consistently decreased when comparing pre-COVID vs COVID-era and in-person vs remote visits. Further studies are needed to clarify whether these decreases represent a reduction in the overuse of tests and medications vs an underuse of indicated testing and prescribing.

## Key Points

**Question:**

Is the transition to remote cardiology ambulatory visits during the COVID-19 pandemic associated with disparities in patient access to care, ordering of diagnostic tests, and/or medication prescribing?

**Findings:**

In this cross-sectional study of 176 781 ambulatory cardiology visits, patients using COVID-era remote visits were more likely to be Asian, Black, or Hispanic individuals, have private insurance, and have cardiovascular comorbidities. Compared with pre-COVID in-person visits, clinicians during COVID-era video and telephone visits had a significantly lower odds of ordering any medication as well as most tests.

**Meaning:**

Remote cardiology clinic visits were used more often by certain traditionally underserved patient groups but were also associated with less frequent testing and prescribing.

## Teaser

This cross-sectional study examines ambulatory cardiology clinic visit data and whether the transition to remote visits during COVID-19 is associated with disparities in patient use of care, diagnostic test ordering, and medication prescribing.

## Introduction

The COVID-19 pandemic has led to an unprecedented shift in ambulatory care from in-person to remote visits.^1^ Changes to federal regulations and Centers for Medicare and Medicaid Services (CMS) reimbursement policies facilitated an increase in the number of Medicare beneficiaries using telemedicine services from 13 000 individuals a week prior to COVID-19 to nearly 1.7 million individuals in the last week of April 2020.^2^ Prior work, often conducted in controlled study settings, has indicated that telemedicine has the potential to improve care for patients, including those with cardiovascular conditions.^3,4,5,6,7^

It remains unknown how this large-scale transition to remote care in the real world has changed clinical practice patterns in cardiology both in terms of the patients who are able to access care as well as the type and quality of care that is being delivered. As there is currently a paucity of formal guidelines specifying best practices for remote cardiology visits, there may be unintended consequences from this new form of care that have yet to be identified.^8,9,10^ We hypothesized that because of the so-called digital divide, certain patient groups such as those who are older, from racial and ethnic minority groups, and with more comorbidities might be less able to use remote care, especially video-based care.^11^ We further conjectured that because of the lack of physical exam, remote visits might result in fewer medication changes and more diagnostic tests such as nuclear stress imaging and brain natriuretic peptide tests.

To test these hypotheses, we characterized the patient demographic characteristics and clinician ordering frequencies of medications and diagnostic tests at the visit level for all remote and in-person ambulatory cardiology visits at our multisite health system in the COVID-era period. We then compared these visits to those from the same period one year before, during the pre-COVID period.

## Methods

### Study Design and Participants

This cross-sectional study protocol was approved by the Cedars-Sinai Medical Center institutional review board. A waiver of participant consent was obtained given that the study used limited identifying information and there was no interaction with participants or impact on their clinical care. We followed the Strengthening the Reporting of Observational Studies in Epidemiology (STROBE) reporting guideline for methods and results.^12^

This study identified all ambulatory cardiology visits at our large multisite urban health system conducted during the periods of April 1, 2019, to December 31, 2019, and April 1, 2020, to December 31, 2020. This included both in-person visits and remote visits from 31 different cardiology clinics in the Los Angeles, California, metropolitan area. Remote visits could consist of either telephone or video appointment. Clinicians selected the video platform used for video visits; these included an electronic health record (EHR) platform (Epic Systems) or third party vendor (ie, Doximity, Zoom, or WebEx). The COVID period of April 1, 2020, to December 31, 2020, was chosen as it was when remote visits were most frequent. The comparator cohort (pre-COVID) from April 1, 2019, to December 31, 2019, was used to minimize seasonal fluctuations in patient composition and ordering practices. Individual patients could have multiple included visits during the study period.

### Data Sources and Variables

For study estimators, we used visit-level encounter information available from the EHR to determine visit type (in-person, video, or telephone), patient demographic characteristics, visit date, number of visits per patient during the study period, scheduled visit duration, clinician, encounter diagnoses, and all diagnostic and medication orders associated with each visit. For study outcomes, we investigated the ordering frequencies of the most common cardiology-specific diagnostic tests including electrocardiograms (ECGs), transthoracic echocardiograms (TTEs), coronary computed tomography angiography (CCTA), nuclear stress imaging, stress echocardiogram, exercise stress ECG, coronary artery calcium scan, cardiac magnetic resonance imaging (MRI), and external ECG monitoring tests (such as Zio patch monitors). Laboratory tests of interest included B-type natriuretic peptide (BNP), lipid panel, complete blood count, metabolic panel, coagulation studies, erythrocyte sedimentation rate (ESR) and C-reactive protein (CRP) tests. We visualized the ordering frequencies of tests and medications by visit type categorized by month alongside local daily COVID-19 case rates in Los Angeles County as reported by the Los Angeles County Department of Public Health.

### Statistical Analysis

Visits were stratified by time period (pre-COVID vs COVID era) and visit type (in-person vs video vs telephone). All ambulatory visits in 2019 were in person, which resulted in 4 main visit types: pre-Covid in-person, COVID-era in-person, COVID-era video, and COVID-era telephone. For each visit type, patient and visit characteristics were expressed as frequency counts and percentages. We compared rates of medication and diagnostic test ordering by visit type. The differences in discrete variables between groups were evaluated by the χ^2^ test. Differences in continuous variables were evaluated using the *t* test or analysis of variance (ANOVA) test for multiple groups.

To adjust for the effects of patient and visit characteristics on ordering patterns, we performed multivariable regression. We used multivariable linear regression to study the association between visit type and the number of diagnostic tests ordered per visit. We used multivariable logistic regression to study the association between visit type and the odds of ordering at least one medication or one test as well as the odds of ordering individual diagnostic and laboratory tests. All multivariable analyses adjusted for patient demographics and visit characteristics that would potentially affect clinician ordering patterns (age, sex, race, insurance status, visit length, coronary artery disease, hypertension, atrial fibrillation or flutter, heart failure, diabetes, valvular disease, and chronic kidney disease) as well as calendar date (month and day) to account for potential changes in practice patterns as the pandemic progressed. Multivariable analyses additionally adjusted for individual clinicians (155 clinicians in total) to account for possible differences in practice patterns across different clinicians. Analyses studying ordering practices over time did not adjust for calendar date, because patients were already stratified by month. All hypothesis testing was 2-sided and results were evaluated with a significance level of α = .05. All analysis was performed with R statistical software version 3.4.1 (R Project for Statistical Computing) from November 2020 to February 2021.

## Results

The study cohort included 176 781 ambulatory cardiology visits, with 87 182 of these being in-person visits in the pre-COVID period, 74 498 COVID-era in-person visits, 4720 COVID-era video visits, and 10 381 COVID-era telephone visits. Among all visits, 79 572 patients were female (45.0%), 127 080 patients were Non-Hispanic White (71.9%), and the mean (SD) age was 68.1 (17.0) years. Although all visits in the pre-COVID cohort were in-person, 10 381 visits (11.6%) in the COVID-era cohort were by telephone and 4720 visits (5.3%) were by video. Comparison of baseline characteristics between the 4 visit types (pre-COVID in-person, COVID-era in-person, COVID-era video, and COVID-era telephone) demonstrated many statistically significant differences, and several clinically meaningful ones (Table 1). Patients seen by in-person and telephone visits were of similar age, whereas those seen by video visit had a significantly younger mean (SD) age (pre-COVID in-person: 67.7 (17.3) years; COVID-era in-person: 69.0 (16.7) years; COVID-era video: 61.1 (16.5) years, COVID-era telephone: 68.4 (16.0) years; *P* < .001 for COVID-era in-person vs video and COVID-era in-person vs telephone). Although patients from underrepresented racial and ethnic groups were seen at similar or slightly lower rates in the COVID-era period, they constituted a larger proportion of remote visits (pre-COVID in-person: 5973 Asian patients [6.9%], 6366 Black patients [7.3%], 4661 Hispanic patients [5.3%] vs COVID-era in-person: 4202 Asian patients [5.6%], 5321 Black patients [7.1%], 3482 Hispanic patients [4.7%] vs COVID-era video visits: 378 Asian patients [8.0%], 390 Black patients [8.3%], 255 Hispanic patients [5.4%] vs COVID-era telephone visits: 810 Asian patients [7.8%], 1073 Black patients [10.3%], 664 Hispanic patients [6.4%]; *P* < .001 for COVID-era in-person vs video and COVID-era in-person vs telephone) (summed data for all underrepresented racial and ethnic groups: 24 934 pre-COVID in-person visits [28.6%] vs 19 742 COVID-era in-person visits [26.5%] vs 3633 COVID-era video visits [30.4%] vs 1435 COVID-era telephone visits [35.0%]; *P* < .001 for all comparisons). A larger proportion of remote visits were also with patients who had private insurance (34 063 pre-COVID in-person visits [39.1%] vs 25 474 COVID-era in-person visits [34.2%] vs 2562 COVID-era video visits [54.3%] vs 4264 COVID-era telephone visits [41.1%]; *P* < .001 for COVID-era in-person vs video and COVID-era in-person vs telephone). Patients seen by remote visit had more cardiovascular comorbidities, including hypertension (37 166 pre-COVID in-person visits [42.6%] vs 31 359 COVID-era in-person visits [42.1%] vs 2006 COVID-era video visits [42.5%] vs 5181 COVID-era telephone visits [49.9%]; *P* < .001 for COVID-era in-person vs telephone), coronary artery disease (24 600 pre-COVID in-person visits [28.2%] vs 20 363 COVID-era in-person visits [27.3%] vs 1265 COVID-era video visits [26.8%] vs 3533 COVID-era telephone visits [34.0%]; *P* < .001 for COVID-era in-person vs telephone), atrial fibrillation or flutter (16 706 pre-COVID in-person visits [19.2%] vs 13 292 COVID-era in-person visits [17.8%] vs 1343 COVID-era video visits [28.5%] vs 2426 COVID-era telephone visits [23.4%]; *P* < .001 for COVID-era in-person vs video and COVID-era in-person vs telephone), heart failure (14 319 pre-COVID in-person visits [16.4%] vs 10 488 COVID-era in-person visits [14.1%] vs 1172 COVID-era video visits [24.8%] vs 2674 COVID-era telephone visits [25.8%]; *P* < .001 for COVID-era in-person vs video and COVID-era in-person vs telephone), and chronic kidney disease (3579 pre-COVID in-person visits (4.1%) vs 3112 COVID-era in-person visits [4.2%] vs 243 COVID-era video visits [5.1%] vs 827 COVID-era telephone visits [8.0%]; *P* < .001 for COVID-era in-person vs video and COVID-era in-person vs telephone). Despite having more comorbidities, patients seen by remote visit had fewer mean (SD) visits overall during the study period (2.33 [2.11] pre-COVID in-person visits vs 2.47 [2.69] COVID-era in-person visits vs COVID-era video visits 1.48 [0.97] vs COVID-era telephone visits 1.51 [1.09]; *P* < .001 for COVID-era in-person vs video and COVID-era in-person vs telephone).

**Table 1.** Patient and Visit Characteristics of Ambulatory Cardiology Clinic Visits During the Pre-COVID and COVID-Era Periods^a^^,^^b^

Characteristic	Visits, No. (%)					*P* value		
	Pre-COVID visits (all in person)	COVID-era visits						
		All	In-person	Video	Telephone	Pre-COVID vs COVID-era	In-person vs video^c^	In-person vs telephone^c^
Overall	87182	89 599	74 498 (83.2)	4720 (5.3)	10 381 (11.6)	NA	NA	NA
Age, mean (SD), y	67.66 (17.32)	68.56 (16.66)	69.04 (16.65)	61.09 (16.49)	68.44 (15.99)	<.001	<.001	<.001
Sex								
Female	38 926 (44.6)	40 646 (45.4)	33 441 (44.9)	1968 (41.7)	5237 (50.4)	.01	<.001	<.001
Male	48 249 (55.3)	48 947 (54.6)	41 051 (55.1)	2752 (58.3)	5144 (49.6)			
Race								
American Indian	180 (0.2)	152 (0.2)	126 (0.2)	7 (0.1)	19 (0.2)	<.001	.01	<.001
Asian	5973 (6.9)	5390 (6.0)	4202 (5.6)	378 (8.0)	810 (7.8)			
Black	6366 (7.3)	6784 (7.6)	5321 (7.1)	390 (8.3)	1073 (10.3)			
Hispanic	4661 (5.3)	4401 (4.9)	3482 (4.7)	255 (5.4)	664 (6.4)			
White	62 264 (71.4)	64 816 (72.3)	54 789 (73.5)	3283 (69.6)	6744 (65.0)			
Other	4408 (5.1)	4383 (4.9)	3618 (4.9)	235 (5.0)	530 (5.1)			
Pacific Islander	122 (0.1)	131 (0.1)	103 (0.1)	7 (0.1)	21 (0.2)			
Unknown	3208 (3.7)	3542 (4.0)	2857 (3.8)	165 (3.5)	520 (5.0)			
Insurance								
Medicaid	1324 (1.5)	1104 (1.2)	821 (1.1)	90 (1.9)	193 (1.9)	<.001	<.001	<.001
Medicare	48 380 (55.5)	46 993 (52.4)	39 567 (53.1)	1891 (40.1)	5535 (53.3)			
Other	1040 (1.2)	851 (0.9)	679 (0.9)	75 (1.6)	97 (0.9)			
Private	34 063 (39.1)	32 300 (36.0)	25 474 (34.2)	2562 (54.3)	4264 (41.1)			
Unknown	2375 (2.7)	8351 (9.3)	7957 (10.7)	102 (2.2)	292 (2.8)			
Visits/patient, mean (SD), No.	2.33 (2.11)	2.56 (2.7)	2.47 (2.69)	1.48 (0.97)	1.51 (1.09)	<.001	<.001	<.001
Visit length, mean (SD), min	28.98 (15.12)	25.51 (13.69)	25.45 (13.76)	28.83 (11.88)	24.46 (13.70)	<.001	.50	<.001
Encounter diagnoses								
Coronary artery disease	24 600 (28.2)	25 161 (28.1)	20 363 (27.3)	1265 (26.8)	3533 (34.0)	.53	.04	<.001
Hypertension	37 166 (42.6)	38 546 (43.0)	31 359 (42.1)	2006 (42.5)	5181 (49.9)	.10	.87	<.001
Atrial fibrillation or flutter	16 706 (19.2)	17 061 (19.0)	13 292 (17.8)	1343 (28.5)	2426 (23.4)	.52	<.001	<.001
Heart failure	14 319 (16.4)	14 334 (16.0)	10 488 (14.1)	1172 (24.8)	2674 (25.8)	.02	<.001	<.001
Diabetes	7809 (9.0)	8456 (9.4)	7040 (9.4)	413 (8.8)	1003 (9.7)	<.001	.65	.02
Valvular disease	19 148 (22.0)	18 051 (20.1)	15 475 (20.8)	617 (13.1)	1959 (18.9)	<.001	<.001	<.001
Chronic kidney disease	3579 (4.1)	4182 (4.7)	3112 (4.2)	243 (5.1)	827 (8.0)	<.001	.001	<.001

Abbreviation: NA, not applicable.

^a^ Pre-COVID period was considered from April 1, 2019, to December 31, 2019. COVID-era was considered from April 1, 2020, to December 31, 2020.

^b^ All comparisons performed using *t* test.

^c^ In-person refers to COVID-era in-person visits.

**Figure 1.**
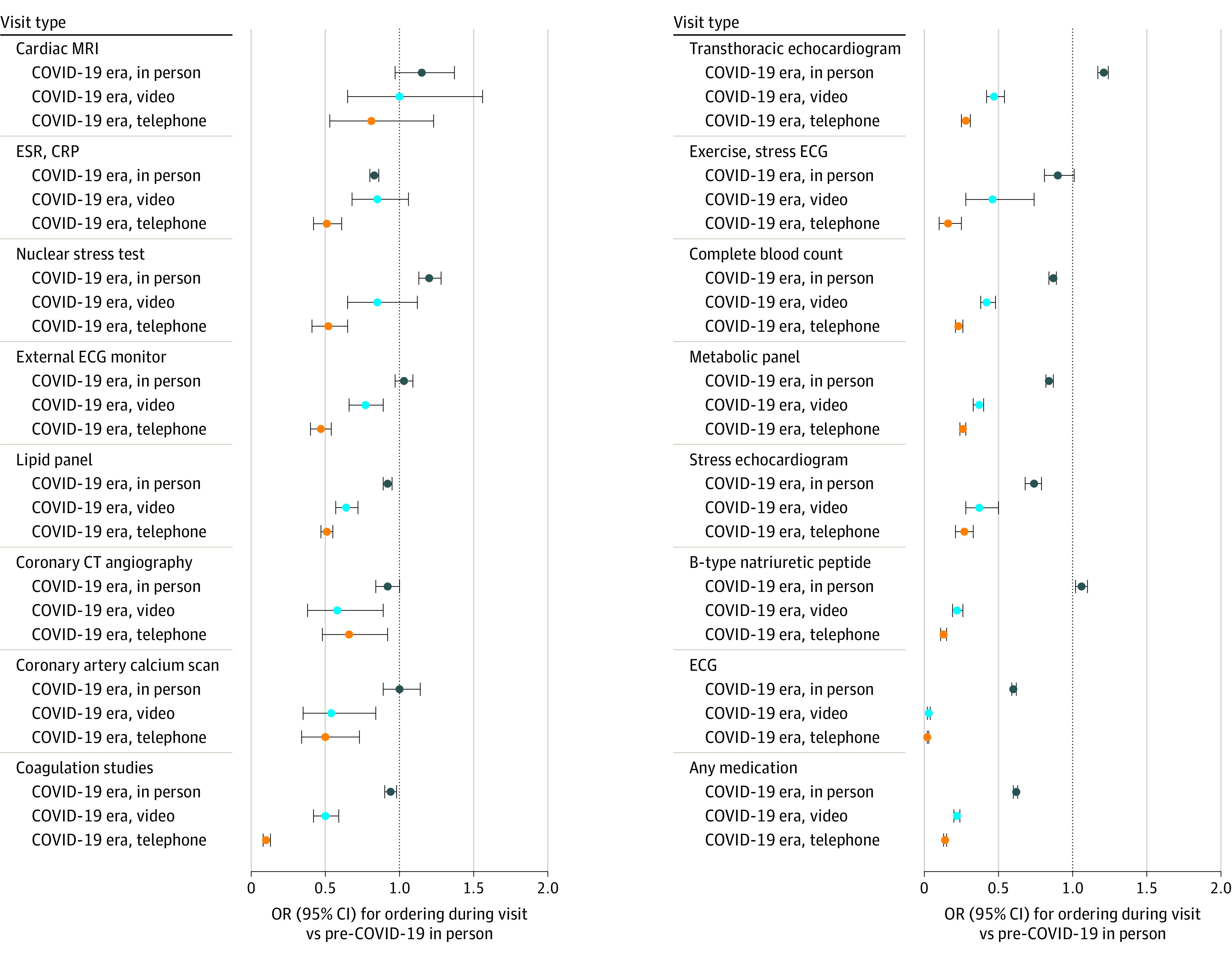
Odds of Placing Specific Orders During Clinic Visit When Compared With Pre-COVID In-Person Visits All estimates were adjusted for age, sex, race, insurance status, clinician, calendar date, visit length, and visit diagnoses (coronary artery disease, hypertension, atrial fibrillation/flutter, heart failure, diabetes, valvular disease, and chronic kidney disease). MRI indicates magnetic resonance imaging; ESR, erythrocyte sedimentation rate; CRP, C-reactive protein; ECG, electrocardiogram; CT, computed tomography.

When comparing ordering practices between pre-COVID in-person, COVID-era in-person, COVID-era video, and COVID-era telephone visits, we found a decrease in the proportion of visits where at least one medication was ordered (Table 2). The proportion of visits where at least one medication was prescribed decreased from more than two-thirds of pre-COVID in-person visits to half of COVID-era in-person visits to only one-third of COVID-era video visits and one-quarter of COVID-era telephone visits. This trend remained true after multivariable adjustment for visit and patient characteristics (pre-COVID in-person: reference, COVID-era in-person: odds ratio [OR], 0.62; 95% CI, 0.60-0.64; COVID-era video: OR, 0.22; 95% CI, 0.20-0.24; COVID-era telephone: OR, 0.14; 95% CI, 0.13-0.15) (Figure 1). This same pattern was seen across nearly all diagnostic and laboratory tests with a stepwise decrease in ordering frequency when comparing in-person to video to telephone visits in the COVID-era period (eg, ECG: OR, 0.60 [95% CI, 0.58-0.62] vs OR, 0.03 [95% CI, 0.02-0.04] vs OR, 0.02 [95% CI, 0.01-0.03]; TTE: OR, 1.21 [95% CI, 1.18-1.24] vs OR, 0.47 [95% CI, 0.42-0.52] vs OR, 0.28 [95% CI, 0.25-0.31]; BNP: OR, 1.06 [95% CI, 1.02-1.10] vs OR, 0.22 [95% CI, 0.19-0.25] vs OR, 0.13 [95% CI, 0.11-0.15]) (Table 2, Figure 1).

**Table 2.** Percentage of Visits During Which Medication or a Test Was Ordered

Medication or Test	Pre-COVID in-person	COVID-era in-person	COVID-era video	COVID-era telephone
Any medication	68.09	55.73	33.64	23.53
ECG	37.57	27.86	1.72	1.05
TTE	14.54	15.12	6.84	3.9
Coronary CT angiography	1.56	1.51	0.49	0.39
Nuclear stress test	2.52	3.17	1.27	0.8
Stress echocardiogram	2.62	1.74	1.1	0.76
Exercise stress ECG	1.09	0.84	0.4	0.21
Coronary artery calcium scan	0.76	0.64	0.47	0.28
Cardiac MRI	0.39	0.34	0.51	0.25
External ECG monitor	4.19	3.56	5.32	1.86
B-type natriuretic peptide	15.37	14.06	4.49	3.81
Lipid panel	18.2	17.32	7.73	6.95
Complete blood count	26.72	23.75	8.52	5.52
Metabolic panel	34.07	29.34	13.69	11.04
Coagulation studies	9.14	7.89	3.43	0.83
ESR, CRP	10.88	10.19	2.22	1.38

Abbreviations: CRP, C-reactive protein; CT, computed tomography; ECG, electrocardiogram; ESR, erythrocyte sedimentation rate; MRI, magnetic resonance imaging; TTE, transthoracic echocardiogram.

**Figure 2.**
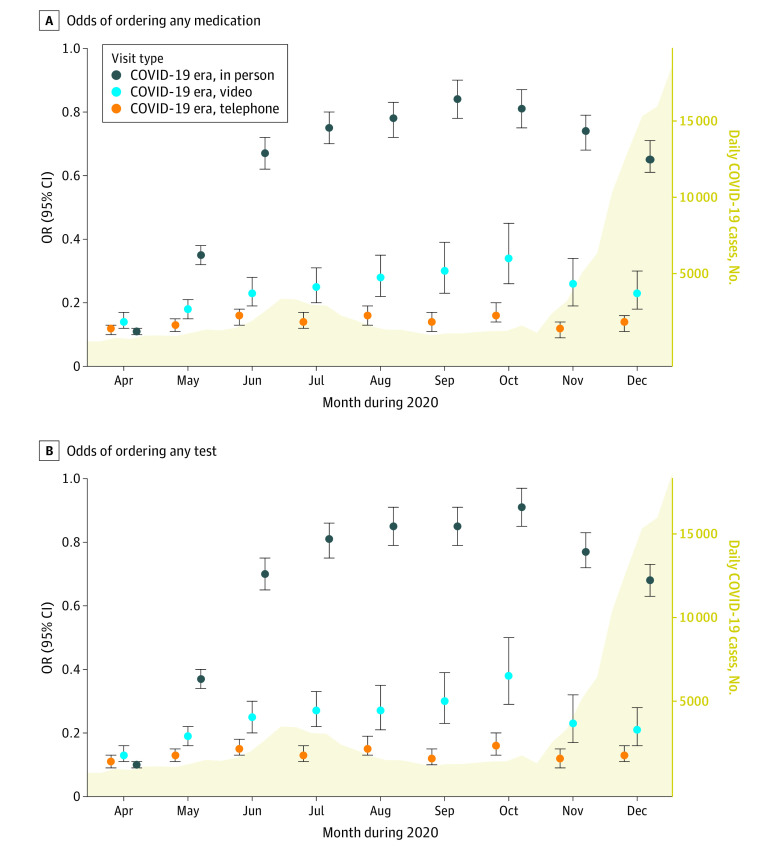
Odds Over Time of Ordering Any Test or Medication During COVID-Era Clinic Visits Compared With Pre-COVID In-Person Visits All estimates were adjusted for age, sex, race, insurance status, clinician, visit length, and visit diagnoses (coronary artery disease, hypertension, atrial fibrillation or flutter, heart failure, diabetes, valvular disease, and chronic kidney disease). Yellow curve represents number of new daily COVID cases in Los Angeles County during the same months.

When visualizing ordering practices over time, it appeared that rates of ordering tests and medications generally went up during months when there were lower COVID-19 daily case numbers and down when COVID-19 case numbers increased (Figure 2). However, in comparison with pre-COVID in-person visits, there continued to be a stepwise decrease in rates of ordering at least one test or medication for COVID-era in-person, COVID-era video, and COVID-era telephone visits across all months (Figure 2).

## Discussion

In an analysis of 176 781 ambulatory cardiology visits, we found significantly higher use of remote cardiology clinic visits among Asian, Black, and Hispanic individuals, those with private insurance, and those with cardiovascular comorbidities. We also identified a stepwise reduction in the ordering frequency of both diagnostic tests and prescription medications when comparing pre-COVID with COVID-era in-person and COVID-era video and COVID-era telephone visits. To our knowledge, this represents the first study to investigate how the rapid and dramatic increase in the use of telehealth is associated with changes in cardiovascular patient care in a real-world setting.

Although the use of telehealth has increased gradually during the preceding decades, its widespread use during the COVID-19 pandemic will likely lay the foundation for remote clinic visits as a more permanent and substantial part of future cardiovascular care.^2,13,14,15^ Numerous studies have demonstrated the benefits of remote care using technologies such as event monitors and smartwatches targeted to specific cardiovascular disease processes such as arrhythmias, heart failure, or hypertension.^3,4,5,6,7^ Although these interventions have been largely studied in isolation, the COVID-era period is the first chance to study whether this assortment of modern telemedicine capabilities can result in accessible and effective virtual care in real-world practice.

The promise of telemedicine has always been tempered by concerns about disparities in access driven by the digital divide. Individuals who are older, have lower income, have less education, an underrepresented racial or ethnic group, live in rural areas, and have more chronic conditions are known to have less access to the internet and therefore possibly telemedicine services, especially video visits which require more technology investment and literacy.^11^ Although our data did not allow us to measure differences in care access, we were able to characterize overall differences in care usage. In our patient cohort, remote visits equally replaced the decrease in in-person patient visits in the COVID-era period. We found several differences in the patient populations that used remote care, but not all of the trends were expected. Consistent with expectations, patients with private insurance, a frequent proxy for high socioeconomic status, made up a larger percentage of both video and telephone visits.^16^ This is consistent with another recent study of primary care clinics serving low income individuals that reported that the shift to telehealth visits resulted in a modest decline in overall patient visits, mostly because of the inaccessibility of video visits for low income populations.^1^ We also found that older patients, which may have more difficulties with telemedicine technology, used video visits less frequently, although the mean (SD) age of patients using video visits was still 61.09 (16.49) years and 68.44 (15.99) years, respectively.^17,18^ However, other disparities that had been previously reported or postulated, such as those associated with race and ethnicity, were not identified.^19^ Although patients from underrepresented racial and ethnic groups were seen at similar frequency in the pre-COVID vs COVID-era periods, they in fact used video and telephone visits more frequently than White patients. We also found that patients using remote visits were more likely to have documented cardiovascular comorbidities, indicating comfort by both patients and clinicians in the delivery of complex care by telephone or video.

Although this study was not designed to explain the observed differences in care utilization by patient groups, several possible reasons may be considered. One explanation may be a difference in perceived risk of attending in-person clinic appointments during the pandemic. Numerous studies highlighted in the press have drawn attention to the enhanced risk of COVID-19 infection among older individuals, people of color, and those with cardiovascular comorbidities, potentially convincing these patients and their clinicians to opt for remote visits.^20,21^ It may also be the case that patients who are older, from underrepresented racial and ethnic groups, or have more medical comorbidities find remote visits to be more appealing because they are comparatively less able to access in-person visits, whether that is because of greater barriers to transportation or scheduling.^22^ Indeed, a higher proportion of people of color work so-called essential jobs and may be less able to leave work to travel to in-person appointments.^23^

We demonstrated that across all patients, there were also significant differences in practice patterns by visit type. Prior concerns have been raised about whether telemedicine visits could lead to the overuse of unnecessary testing and the overprescription of medications.^24^ One could imagine, for example, that the lack of physical exam during remote visits with heart failure patients could result in the increased ordering of echocardiograms or lab tests such as BNP. Interestingly, we found the opposite to be true, with a decrease in the frequency of all diagnostics testing with video and telephone visits. The proportion of visits where at least one medication was prescribed decreased from more than two-thirds of pre-COVID in-person visits to half of COVID-era in-person visits to only one-third of COVID-era video visits and one-quarter of COVID-era telephone visits. These data are noteworthy because patients seen by remote visits had more cardiovascular comorbidities and were therefore more likely to require guideline-recommended medical therapies.

A portion of the decreased testing may be explained by reduced access, as many of these tests, such as lab tests, ECGs, echocardiograms, and stress tests, are often performed in the same facility and at the same time as the in-person clinic appointment. Some of the differences in testing may also be associated with shortcomings inherent to remote care such as a poorer understanding of the patient’s clinical picture because of decreased communication clarity and the inability to perform comprehensive physical examinations.^25^ A clinician may not think to order specific tests if not cued by certain findings on history of physical exam. This could be why there was still a further decrease in ordering frequency when comparing telephone with video visits, because telephone visits often provide even less information than video visits given the lack of a visual interface with the patient. The changes in testing, in turn, may have then affected prescribing practices. For example, reduced lab testing to monitor kidney function and electrolytes or lack of clinic-measured vital signs may have hindered clinical decision-making around the initiation and up-titration of frequently used cardiac medication such as beta blockers, ACE-inhibitors, and angiotensin receptor blockers. Further research investigating whether certain medication classes were more affected would be helpful.

Lastly, we studied the differences in ordering practices over time. It could be argued that the observed decreases in testing and prescribing were because clinicians were initially waiting for the COVID-19 pandemic to subside before advancing medical care. Across all visits, we found that ordering practices appeared to change in association with the severity of the local COVID-19 case incidence, but we found that the differences between in-person, video, and telephone visits persisted across nearly every month of the 9-month period of study. Nevertheless, additional follow-up studies after the pandemic has subsided are required to determine how long the decrease in diagnostic testing and medication prescription endures and whether it will have a significant impact on clinical outcomes.

### Limitations

Several limitations to this study merit consideration. Although the strengths of this study include the large number of ambulatory visits from our multicenter institution and the completeness of the data, we studied visits from one medical system and geographical location. Our patient population was largely insured, primarily by private insurance or Medicare, and lived in an urban center, which may not capture certain patient populations most associated with adverse outcomes of the digital divide. Despite this, the diversity of our patient population, including by age, sex, and race contribute to the greater generalizability of our findings. There were also likely complex interactions that arose from both the COVID-19 pandemic as well as the change from in-person to remote visits. Deciphering the degree to which each of these factors contributed to changes in care utilization and practice patterns is difficult and we have attempted to disentangle these associations by limiting some comparisons across visit types to only the COVID-era (ie, COVID-era in-person vs COVID-era video vs COVID-era telephone) as well as by studying practice patterns over time to see if there were observable differences as the pandemic waned. As this is not a randomized study, we are unable to determine the degree to which there was bias from both patients and clinicians as to the selection of which patients were seen by in-person or remote visits. We did find that patients seen by remote visits had more medical comorbidities, which would suggest that the decrease in medication and diagnostic ordering with remote visits was not simply due to less sick patients being seen remotely. However, future prospective studies will be helpful for confirming our findings.

## Conclusions

The rapid and large scale transition from in-person to remote cardiovascular care during the COVID-19 pandemic has important implications for patient access to care as well as clinician practice patterns. In this large study of ambulatory cardiology visits, we found that during the height of the COVID-19 pandemic, remote cardiovascular care was more frequently accessed by patients who were younger, from underrepresented racial and ethnic groups, had private insurance, or had more cardiovascular comorbidities. There was a stepwise decrease in the ordering frequency of both diagnostic testing and medications when comparing pre-COVID in-person to COVID-era in-person to COVID-era video to COVID-era telephone visits. As a substantial proportion of future cardiology ambulatory care will likely continue to be delivered through remote visits, these changes in care access and practice patterns will have substantial ramifications with regards to both the efficacy and cost of future cardiovascular care.
